# Dynamic Effects of Topoisomerase I Inhibition on R-Loops and Short Transcripts at Active Promoters

**DOI:** 10.1371/journal.pone.0147053

**Published:** 2016-01-19

**Authors:** Jessica Marinello, Stefania Bertoncini, Iris Aloisi, Agnese Cristini, Guidantonio Malagoli Tagliazucchi, Mattia Forcato, Olivier Sordet, Giovanni Capranico

**Affiliations:** 1 Department of Pharmacy and Biotechnology, University of Bologna, Bologna, Italy; 2 Cancer Research Center of Toulouse, INSERM UMR1037, Toulouse, France; 3 Center for Genome Research, Department of Life Sciences, University of Modena and Reggio Emilia, Modena, Italy; Florida International University Bimolecular Sciences Institute, UNITED STATES

## Abstract

Topoisomerase I-DNA-cleavage complexes (Top1cc) stabilized by camptothecin (CPT) have specific effects at transcriptional levels. We recently reported that Top1cc increase antisense transcript (aRNAs) levels at divergent CpG-island promoters and, transiently, DNA/RNA hybrids (R-loop) in nuclear and mitochondrial genomes of colon cancer HCT116 cells. However, the relationship between R-loops and aRNAs was not established. Here, we show that aRNAs can form R-loops in N-TERA-2 cells under physiological conditions, and that promoter-associated R-loops are somewhat increased and extended in length immediately upon cell exposure to CPT. In contrast, persistent Top1ccs reduce the majority of R-loops suggesting that CPT-accumulated aRNAs are not commonly involved in R-loops. The enhancement of aRNAs by Top1ccs is present both in human colon cancer HCT116 cells and WI38 fibroblasts suggesting a common response of cancer and normal cells. Although Top1ccs lead to DSB and DDR kinases activation, we do not detect a dependence of aRNA accumulation on ATM or DNA-PK activation. However, we showed that the cell response to persistent Top1ccs can involve an impairment of aRNA turnover rather than a higher synthesis rate. Finally, a genome-wide analysis shows that persistent Top1ccs also determine an accumulation of sense transcripts at 5’-end gene regions suggesting an increased occurrence of truncated transcripts. Taken together, the results indicate that Top1 may regulate transcription initiation by modulating RNA polymerase-generated negative supercoils, which can in turn favor R-loop formation at promoters, and that transcript accumulation at TSS is a response to persistent transcriptional stress by Top1 poisoning.

## Introduction

Topoisomerase I (Top1) is a fundamental nuclear enzyme regulating DNA superhelicity, and its activity is required for a proper progression of transcription and replication machineries in mammalian cells. Enzyme catalysis can essentially be divided into four steps: substrate binding, DNA cleavage, controlled strand rotation, and DNA resealing. Anticancer Top1 poisons, such as camptothecin (CPT), inhibit the last step by binding at the interface of Top1-DNA complexes (Top1cc) at the DNA cleavage site and leaving the enzyme covalently bound to DNA [1]. Top1cc is intrinsically reversible, however it can lead to irreversible double-stranded DNA breaks when a collision occurs with replication forks or elongating RNA polymerases (RNA Pol)[2,3]. The replication-dependent irreversible DNA damage is commonly considered the molecular basis of CPT cytotoxicity and antitumor activity, as it can act as a potent inducer of cancer cell apoptosis[4].

In addition to cell killing activity, Top1 poisons have specific effects at transcriptional levels that may impact gene expression profiles of normal and/or cancer cells contributing to drug therapeutic outcomes[2,5,6,7,8,9]. Recently, treatments with Top1 poisons have been shown to de-repress the paternal Ube3A allele in an Angelman disease murine model[10]providing an interesting case in which CPT derivatives can permanently change the expression of a specific gene in mammalian cells. Thus, understanding the mechanisms of Top1 regulation of gene expression and the interference of Top1 inhibitors with them can provide significant insights to discover new anticancer therapeutics.

Top1 is a very active enzyme at transcribed regions[11,12,13]. A main role has been suggested to be the regulation of DNA superhelicity at intermediately-active genes as its inhibition by CPT increased local negative supercoils at corresponding promoters[14]. Interestingly, we have demonstrated that CPT impacts transcription regulation with characteristic and specific effects on RNA Pol II recruitment and pausing, nucleosome density and promoter-associated antisense RNA levels [2,5,15]. In particular, a genome-wide analysis revealed that CPT increases antisense transcripts levels at active divergent CpG-island promoters (CGI) in a manner dependent on Top1cc formation [6]. Whether the increase in negative DNA supercoils at promoters is mechanistically linked to the specific effects on RNAPolII and antisense transcripts was left to be defined.

Top1 silencing is known to increase non-B DNA structures, such as R-loops, that are prone to DNA damage and genome instability[16,17,18]. R-loops are three-strand structures constituted by a DNA-RNA hybrid duplex and a displaced DNA strand. Stable R-loops exist in living prokaryotic and eukaryotic cells at origin of replication where they have a role in the regulation of replication initiation[19,20,21]. R-loops also constitute a necessary step of the immunoglobulin recombination mechanism as they form at IgG class switch regions where they can extend over a kilobase[22,23]. Moreover, differential stabilization of R-loops could influence gene expression in many organisms. For instance, R-loop structures allow the presence of the substrate for a single-strand DNA binding protein that represses the expression of COOLAIR ncRNA in Arabidopsis [24]. In addition, R-loops can be enriched over human CGI and involved in maintaining their hypomethylated state [25]. Gene mutations affecting nucleic acid degradation have recently been shown to cause global DNA hypomethylation and R-loop accumulation in fibroblasts of patients with autoimmune disorders [26]. Interestingly, Top1 poisoning by CPT can induce specific double-stranded DNA cleavage in post-mitotic cells that can be suppressed by the overexpression of RNaseH1, suggesting the involvement of transcriptionally-linked R-loops in CPT induction of DNA damage[27]. Increased R-loop levels by CPT have been shown at specific loci such as Angelman imprinting locus and Fragile X syndrome site [28,29]. With a time course analysis in human colon cancer cells by confocal cell microscopy, we showed that CPT effects on R-loops are highly dynamic as it triggers a transient increase of global R-loops in the nuclear and mitochondrial genomes [6]. However, the genomic location of such dynamic R-loop structures and their relationships with aRNA have not been established yet.

Therefore, we have here asked whether CPT-increased aRNAs can form R-loops at specific divergent promoters. Our findings show that R-loops form at divergent promoters and Top1 inhibition by CPT can dynamically modulate their formation. In addition, antisense transcripts are likely increased by persistent Top1ccs due to the inhibition of their degradation, with a simultaneous accumulation of truncated sense transcripts at active promoters.

## Materials and Methods

### Cell lines

The cancer cell lines HCT116 and N-Tera-2 cl.D1 were purchased from ATCC (LGC Standards S.r.l., Milan, Italy) and were grown in DMEM medium with 10% fetal bovine serum (Carlo Erba, Milan, Italy). Cells were maintained at 37°C in a humidified incubator containing 20% O_2_ and 5% CO_2_. Cell line identity was certified with Cell ID System (Promega) by BMR Genomics Srl (Padova, Italy). Immortalized WI-38 human embryonic fibroblasts (WI-38hTERT) were obtained from Carl Mann (CEA, Gif-sur-Yvette, France) and Estelle Nicolas (Université de Toulouse, Toulouse, France) [30]. WI-38hTERT cells were cultured in modified Eagle's medium (MEM) supplemented with 10% fetal bovine serum, 1 mM sodium pyruvate, 2 mM l-glutamine and 0.1 mM MEM non-essential amino acids (Life Technologies). For quiescence induction, cells were washed twice with serum-free medium and grown in MEM with 0.2% serum for 72 h.

### Drugs and cell treatments

CPT and flavopiridol were purchased from Sigma-Aldrich. ATM inhibitor (KU55933) and DNA-PK inhibitor (NU7441) were obtained from Calbiochem and Tocris respectively. Serum-starved or exponentially growing cells were exposed to 10μM CPT for the indicated time at 37°C, unless specified otherwise. In case of co-treatments, cells were previously incubated with various inhibitors for 1 h before the addition of CPT to the medium for further 4 hours.

### RNA extraction and cDNA preparation

Total cellular RNA was purified with the acid phenol method[15,31]and quantified by UV absorbance. After verifying its quality on a 1% agarose gel, 1 μg of total RNA was used to prepare cDNA using SuperScript III (Invitrogen) following the manufacturer’s instruction. Random (N6) and poly(T) primers were used for total RNA retrotranscription. Reactions included a 25°C pre-annealing step for 5 min, and then retrotranscription was performed at 50°C for 50 min.

### Quantitative real-time PCR

Real-time PCR were performed using Applied Biosystems StepOne and SYBR Select Master Mix for CFX (Applied Biosystems). Quantification and melting curve analyses were performed using StepOne Software v2.2.3 as indicated by the supplier. Specificity of PCR products was routinely controlled by melting curve analysis and agarose gel electrophoresis.

### Expression and purification of MBP-RNase H1 (D145N)

Protocol was obtained from Ginno et al 2012 [25]and the plasmid expressing a mutated and inactive RNaseH1 was kindly provided by F. Chedin (University of California, DAVIS). In particular,transformed Rosetta 2(DE3) cells of E. coli were inoculated in LB medium supplemented with 2 g/L of glucose, 100 μg/mL of ampicillin, and 30 μg/mL of chloramphenicol. After induction for 4 hours with IPTG, cells were pelletted and lysed using for 80 ml of cell culture, 1.2 ml of lysis buffer [200 mM NaCl; 20 mM Tris-HCl (pH 7.5); 1 mM EDTA; 10 mM DTT; Aprotinin 2μg/ml; Leupeptin 1μg/ml; Pepstatin 1μg/ml; PMSF 1 mM and Lysozym 200μg/ml]. Lysate was sonicated for 10 min with 30 sec on/off cycles and finally centrifuged at 4°C, 14000g for 20’. The isolation of MBP-fusion protein was performed with Amylose Magnetic Beads (New England Biolabs). In particular 100 μl of beads suspension were equilibrated twice with 500 μl of MBP column buffer [200 mM NaCl; 20 mM Tris-HCl (pH 7.4); 1 mM EDTA and 1 mM DTT] thus incubated with 500 μl of cell culture supernatant at 4°C with agitation for 1 hour. Supernatant was discarded and beads washed three times with 500 μl of MBP column buffer. The purified MBP-RNase H1 (D145N)waseluted from the beads twice with 50 μl of MBP column buffer containing 10 mM maltose for 10 minutes at 4°C with agitation. Alternatively MBP-RNase H1 (D145N) was purified using Amylose Resin (New England Biolabs). In particular, 1 ml resin was poured in a column and washed with 5 column volumes of MBP column buffer. Crude extract was loaded after 1:1 dilution with MBP column buffer and the resin was washed with 12 column volumes of MBP column buffer. Elution of the fusion protein was performed with 5 volumes MBP column buffer containing 10 mM maltose. Purified protein was concentrated with a centrifugal filter unit (Millipore).

### DRIVE (DNA:RNA In Vitro Enrichment)

The procedure was performed as in Ginno et al[25]. In particular, lysis of ~4x106 NTera-2 cl.D1 cells was performed with 1.6 ml TE-SDS Lysis Buffer [10 mM Tris-HCl (pH 8.0); 1 mM EDTA; 0.5% SDS] at 37°C for 5 min. Proteinase K was then added (125 ng/μl) and samples incubated for 5 h at 37°C. An equal volume of phenol (pH 8.0) was added and the samples were mixed gently, thus centrifuged at 3000g for 2 min. The upper phase was collected and an equal volume of chloroform/isoamylic alcohol (24:1) was added to it, then mixed gently and centrifuged again at 3000g for 2 min. Genomic DNA was precipitated with 2.5 volumes of ethanol 100% and 1/10 volume of NaOAc 3M (pH 5.2), in presence of glycogen. Using a hooked glass rod, DNA were spooled out and washed several times with 70% EtOH. Genomic DNA was resuspended in 500 μl of TE buffer [10 mM Tris-HCl (pH 8.0); 1 mM EDTA] avoiding vortexing to preserve RNA/DNA hybrids. Genomic DNA was then digested O/N at 37°C in TANGO BUFFER 2X with 2 mM Spermidine and a cocktail of restriction enzymes: 20 U EcoRI; 20 U Xbal; 20 U Hind III; 20 U SspI and 20 U BsrG1 (ThermoFisherScientific). Each sample was then splitted in three: 1,5 μg were used as input, 1,5 μg were incubated for 2 hours at 37°C with 10U of RNase H1 and 1,5 μg were incubated for 2 hours at 37°C without RNase H1 (Life Technologies). Samples incubated with or without RNaseH1 were added of 50 μl of Binding Buffer 10x [100 mM Na_2_PO_4_ (pH 7.0); 1.4 M NaCl; 0.5% Triton X-100], MBP-RNase H1 D145N (w/w ratio according titration) and TE buffer (pH 7.4) to final volume of 500 μl. RNase H1 D145N was allowed to bind specifically for 2 hours at 4°C on agitation. 50 μl/sample Amylose Magnetic Beads (New England Biolabs) were equilibrated twice with 500 μl of MBP column buffer [200 mM NaCl; 20 mM Tris-HCl (pH 7.4); 1 mM EDTA and 1 mM DTT], then incubated with 500 μl of reaction mix at 4°C with agitation for 75 min. Supernatant was discarded and beads washed three times with 500 μl of MBP column buffer. The purified MBP-RNase H1 (D145N) bound to RNA/DNA hybrid waseluted from the beads twice with 100 μl of MBP column buffer containing 10 mM maltose for 10 minutes at 4°C with agitation. TE (pH 7.4) and SDS (final concentration 0.5%) were added to a final volume of 250 μl. 140 μg of Proteinase K were added and samples incubated at 55°C for 45 min. Samples, included inputs, were thus brought to 300 μl final volume with BDW and phenol/chloroform extraction was performed. To allow precipitation 2,5 volumes of ethanol 100%, 1/10 volume of NaOAc 3M (pH 5.2) and glycogen were added and samples were incubated O/N at -20°C. For data analysis, RNA/DNA hybrid enrichment of each sample is calculated as “% of Input” after subtracting the background signal, as determined by the same sample treated with RNaseH1 before DRIVE precipitation. Then the enrichment value is normalized against the 2-min CPT sample of the RPL13A amplicon of the same experiment.

### Overexpression of wt and mutated RNase H1

Twenty-four hours after seeding (300,000 cells in each well of a 12-wells plate), HCT116 cells were transfected with plasmids overexpressing wt (pRH1) or mutated RNaseH1 (pRH1-D145N), gently furnished by F. Chedin (University of California, DAVIS), using Lipofectamine 2000 (Life Technologies) following manufacturer’s instruction. Twenty-four hours after transfection, the medium was replaced with fresh one and, after additional 24 hours, the drug treatment was started. Finally cells were lysed either for protein extraction and western blot analysis or for RNA extraction and antisense level quantification.

### Bioinformatics analysis

Due to update versions of specific softwares for RNA-Seq analysis, we re-align raw data of previous published experiments[6] to improve previous results. Then, we checked the quality of each sequenced sample using FastQC (http://www.bioinformatics.babraham.ac.uk/projects/fastqc/).Low-quality sequenced bases were filtered out using Trimmomaticv0.33[32]. The bisulfite-treated paired reads were then mapped twice to the hg19 human genome assembly in which all the cytosines were mutated in thymines (CT-hg19) or all the guanine in adenine (GA-hg19) to identify the strand originating the sequence fragment. Sequences coming from positive strand transcripts align to the CT-hg19, whereas negative strand transcripts align to the GA-hg19. The read alignment was carried out with Bowtie2 v2.2.0[33] and TopHat v2.0.10[34,35] to identify known transcripts. For each of the four experiments, reads that aligned to both CT-hg19 and GA-hg19 were discarded. Cufflinks package v2.2.0 [36] was used to assemble and identify novel transcripts, using modified reference genomes (CT-hg19 and GA-hg19). Gene expression levels were estimated in FPKM units (expected number of Fragments Per Kilobase of transcript sequence per Millions of sequenced nucleotides) using Cufflinks. Sense tags distribution, along non-overlapping Refseq genes, were analyzed in a region from 2000 bases upstream the TSS (Transcription Start Site) to 2000 bases downstream the TES using NGSplot software v2.41[37].

## Results

### Promoter-associated aRNAs form R-loops in untreated cells

**Fig 1:**
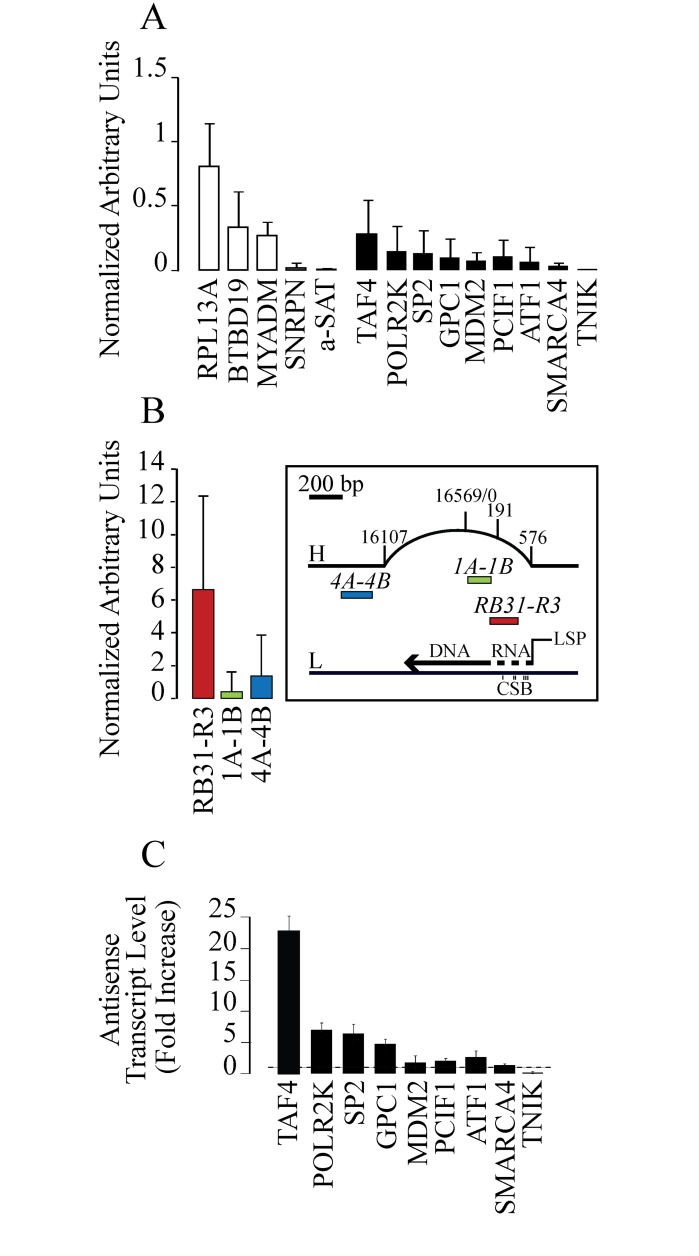
R-loop formation at the studied genomic and mitochondrial regions in untreated control N-TERA-2 cells. DNA enrichment of each sample is subtracted of the enrichment value of the same sample treated with RNase H1 before DRIVE precipitation. Then the enrichment value is normalized against the 2-min CPT sample (see Fig 2) of the RPL13A amplicon of the same experiment. Values are means ±SEM of two to four independent experiments. The data show a higher SEM than commonly published as we report median values of several experiments and not a single representative one. (A) DRIVE assay was performed to determine R-loop levels downstream TSS (white bars) and upstream TSS (black bars). Three negative loci for R-loop formation are also reported (SNRPN, a-SAT, TNIK). (B) Mitochondrial DNA was analyzed with DRIVE assay. Three regions of interest were selected: red for the r-loop forming region (RB31-R3), green for the D-loop region (1A-1B) and blue for the non-D-loop region (4A-4B). Map on the right of the panel shows the heavy (H) and the light (L) strands of mitochondrial DNA, with the three Conserved Sequence Blocks (CSB) and the studied regions (in red, green and blue respectively). (C) Antisense transcription after CPT treatment in N-TERA-2 cells. Promoter-associated antisense transcripts were evaluated by rtqPCR after 4 hours CPT treatment at 10 μM. PCR determinations were normalized to cytochrome b mRNA and to untreated cells (dotted line). Values are means +/− SEM of two determinations from at least two independent experiments.

Both immunofluorescence imaging and RNaseH1 overexpression experiments have demonstrated previously that CPT-trapped Top1ccs induce a significant increase of R-loop structures that can likely mediate drug-induced genome instability [6,27]. However, the genomic sites of such R-loops are not known. Here, we used the DRIVE technique [25,38]to determine whether or not R-loops form at divergent promoters selected on the basis of previous observations of increased aRNAs by camptothecin[6]in human N-TERA-2 cells. To measure the R-loop-specific signal, each sample was split into two parts, one of which was treated with RNase H1 before DRIVE precipitation. Then we calculated the R-loop-specific DNA enrichment of each sample after subtracting the enrichment value of the sample treated with RNase H1 to the not treated (see representative enrichments for untreated and RNase H1-treated samples in Fig A in S1 File). We analyzed R-loop positive and negative loci [25] to check the DRIVE method. Fig 1A (white bars) shows that we could clearly discriminate between regions known to form (RPL13A, BTBD19 and MYADM) and those depleted of (SNRPN and a-Sat) R-loops as a significant recovery is shown at the former but not at the latter loci. Moreover, we determined R-loop levels at the D-loop region of mitochondrial genome (mtDNA), where a well-known and stable R-loop is implicated in replication priming of mtDNA[39]. Of the three selected regions (Fig 1B), the RB31-R3 amplicon corresponds exactly to the R-loop-forming site while 1A-1B and 4A-4B correspond to a D-loop and a more distant regions, respectively. The two non R-loop-forming regions show a recovery significantly lower as compared with RB31-R3 (Fig 1B). Thus, the data showed that we could accurately detect R-loops in mitochondrial and nuclear genomes.

Next, we asked whether R-loops may form upstream to the Transcription Start Site (TSS) of divergent promoters under physiological conditions of cell growth. To that purpose, promoters were selected on the basis of their ability to show an increase of aRNAs after Top1 inhibition by CPT in HCT116 cells [6] and in N-TERA2 cells (Fig 1C). Of the studied promoters, 8 (TAF4, POLR2K, SP2, GPC1, MDM2, PCIF1, ATF1, SMARCA4) showed an increase of aRNA and 1 (TNIK) did not (Fig 1C)[6]. We could readily determine R-loops at all positive promoters, though at different levels, whereas no signal was present at the TNIK promoter (Fig 1A). Even though R-loop levels at the studied divergent promoters are lower than mtDNA sites and other positive controls (RPL13A, BTBD19 and MYADM), they are consistently higher than negative controls (SNRPN, a-Sat and TNIK). In addition, the studiedsites correspond topromoter regions upstream to the TSS, wheretranscription rates are markedly lower than mRNA regions[6], whereasRPL13A, BTBD19 and MYADM regions correspond to thefirst intron of the corresponding pre-mRNA. As these R-loops are likely associated with transcription [25], the results suggest that R-loop formation upstream to TSS reflects the lower aRNA transcription levels as compared with mRNA levels. Thus, taken together, the results show that aRNAs of the studied divergent promoters are able to form R-loopsin untreated cells that are likely in the opposite orientation as comparedwith mRNAs transcription. Promoter-associated antisense R-loops can be transiently stabilized by CPT and then markedly reduced with longer treatment times

**Fig 2:**
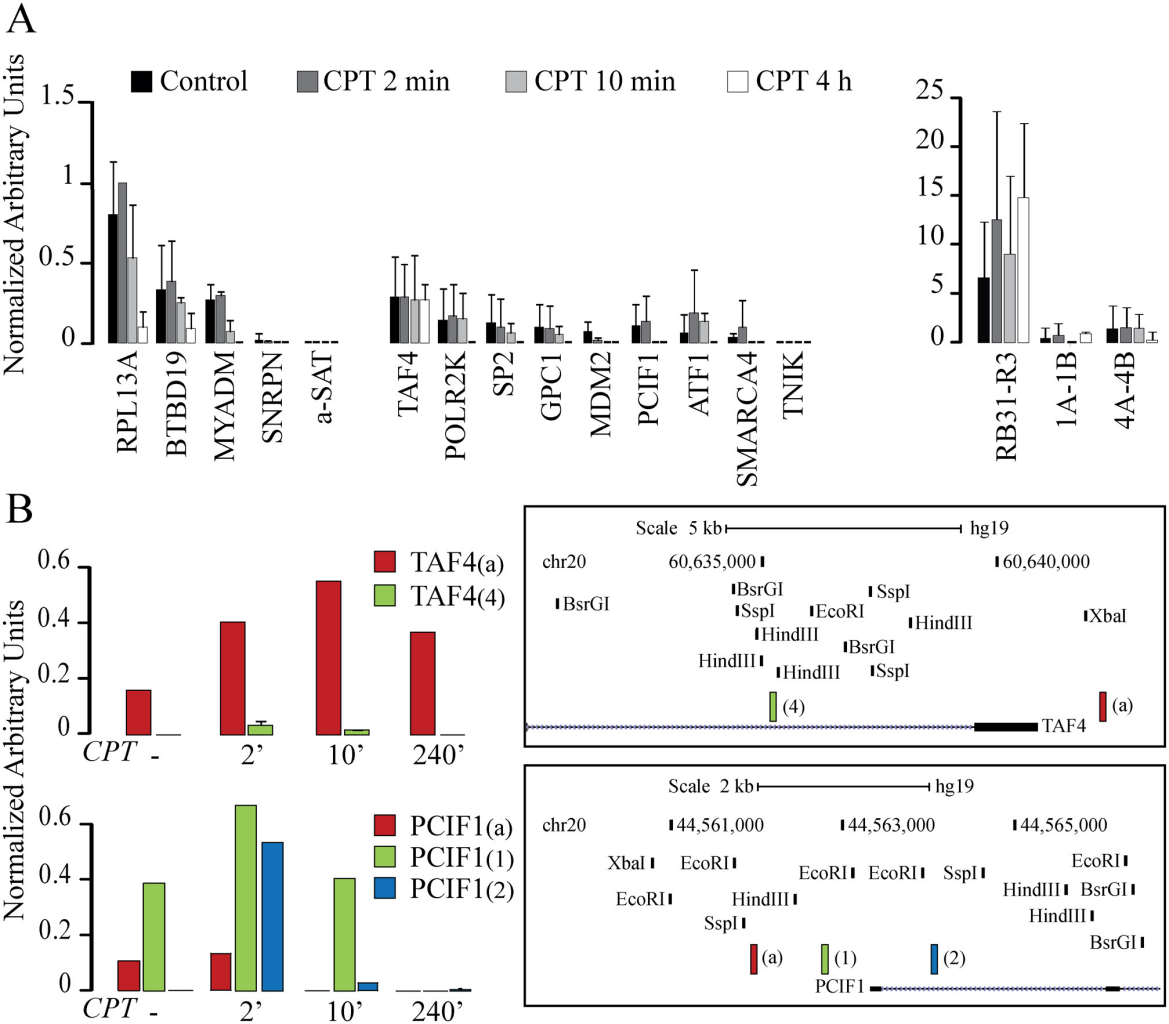
R-loops are transiently stabilized and extended by Top1 inhibition by CPT. R-loop levels as determined by DRIVE at selected genomic regions and active promoters. DNA enrichment of each sample is subtracted of the enrichment value of the same sample treated with RNaseH1 before DRIVE precipitation. Then the enrichment value is normalized against the maximum enrichment value (2-min CPT sample of the RPL13A amplicon) obtained in the same experiment. Values are means ±SEM of two to four independent experiments. (A) R-loop levels at active promoters (on the left) and mitochondrial regions (on the right) after 2 min, 10 min and 4 hours of treatment with 10μM CPT. Control cells are as reported in Fig 1A and 1B to better appreciate variation after drug treatment. (B) R-loop formation at sites close to transcription start site of TAF4 and PCIF1 genes in a time course experiment of CPT treatments. Genome browser views show the genomic localization of the analyzed regions (right panel). DNA digestion with a cocktail of restriction enzymes guarantee the studied regions are properly separated. One representative experiment for each locus is here reported.

Next, we have investigated whether Top1 inhibition by CPT increases R-loop levels at the studied promoters and control regions. We found that the R-loop signal is maintained up to 4 hours of CPT treatment at the mitochondrial replication origin (Fig 2A). Even mitochondrial non-R-loop-forming regions show recoveries unchanged by CPT and significantly lower than the R-loop-forming region. The observed missing effect could be due to the fact that CPT does not target efficiently mitochondrial Top1, due to the limited permeability of mitochondria to non-cationic molecules and because the drug is readily inactivated at alkaline pH [40].

A dynamic pattern was instead observed for R-loops at the transcribed RPL13A, BTBD19 and MYADM nuclear loci. Here, R-loops are slightly increased after short times (2 and 10 minutes), whereas they are markedly reduced after longer time of treatment (4 hours) (Fig 2A). Similarly, antisense R-loops at the studieddivergent promoters show to be somewhat increased atshort treatment times whereas they are completely lost after 4 hours of treatment, with the exception of TAF4 promoter (Fig 2A). Thus, Top1 inhibition by CPT can stabilize antisense and sense R-loops at active divergent promoters but only for a short time.

Thus, it was of interest to determine whether CPT treatment could also affect the length of the DNA-RNA hybrid. Then, we investigated close regions at two of the studied promoters: TAF4 and PCIF1(Fig 2B). These two promoters were chosen as theypresent GC skew segmentsthat can be prone to R-loop formation[38]. Interestingly, at short times of treatment, CPT determines an extension of R-loops or formation of new R-loops, as after 2 minutes the R-loop signal was also detected in TAF4_(4)_ and PCIF1_(2)_ amplicons (Fig 2B). As after CPT treatment R-loop signal is detected in regions where it was not detected before treatment, the findings show that Top1ccs can likely extend and modulate the length of DNA-RNA hybrids at these active promoters.

Taken together, our findings show that following a short time of treatments, CPT can increase R-loops while at longer times the drug almost fully abolishes R-loops at the studied active promoters. The results also showthat after 4 hours of CPT treatment, the accumulated aRNAs are not involved in R-loop structures. As CPT interferes with Top1 activity, the findings clearly show that Top1 can modulate R-loop formation likely by controlling the levels of negative supercoils at promoter regions[14].

As CPT inhibits transcription elongation with high efficacy and R-loops are formed by newly synthesized RNAs, the marked reduction of R-loops at active regions is likely due to persistent inhibition of transcription by CPT. Interestingly, our data show exceptions to that as R-loops can persist for long times of CPT treatment at certain genomic loci (TAF4).

**Fig 3:**
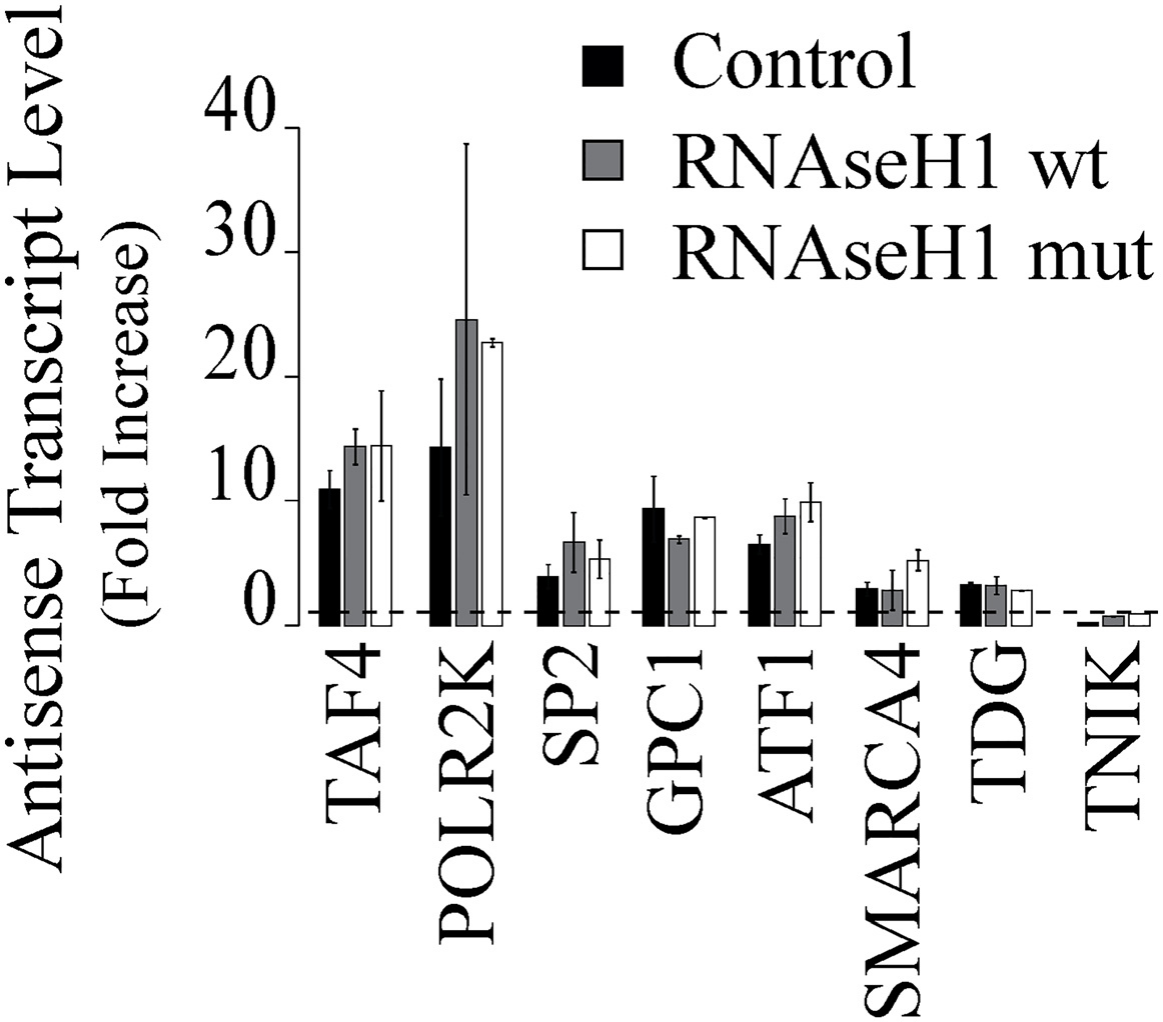
Antisense transcript levels in HCT116 cells overexpressing wt or mutated RNaseH1. Promoter-associated antisense transcripts were evaluated by rtqPCR after 4 hours CPT treatment at 10μM in cells overexpressing a wt or a mutated RNaseH1. PCR determinations were normalized to cytochrome b mRNA and to untreated cells (dotted line). Values are means +/− SEM of two determinations from at least two independent experiments.

To additionally confirm data obtained by DRIVE at long treatment time, i.e. that antisense RNAs are not stably employed in R-loop tertiary structures, we transfected HCT116 cells with a wt and a mutated not catalytically active RNaseH1, the enzyme that specifically catalyzes the cleavage of RNA in a RNA:DNA duplex, and we successively evaluated if the level of those transcripts were modified by overexpression. Fig 3 shows that neither the expression of a wt form of RNaseH1 nor that of a mutated form, significantly modify the level of antisense RNAs after 4 hours of CPT treatment.

The results therefore support the above data obtained by DRIVE and further show that persistent Top1ccs accumulate aRNAs in the cells without forming stable R-loops.

### Antisense transcripts are increased by CPT in human resting normal WI38 cells and are dependent on ongoing transcription

**Fig 4:**
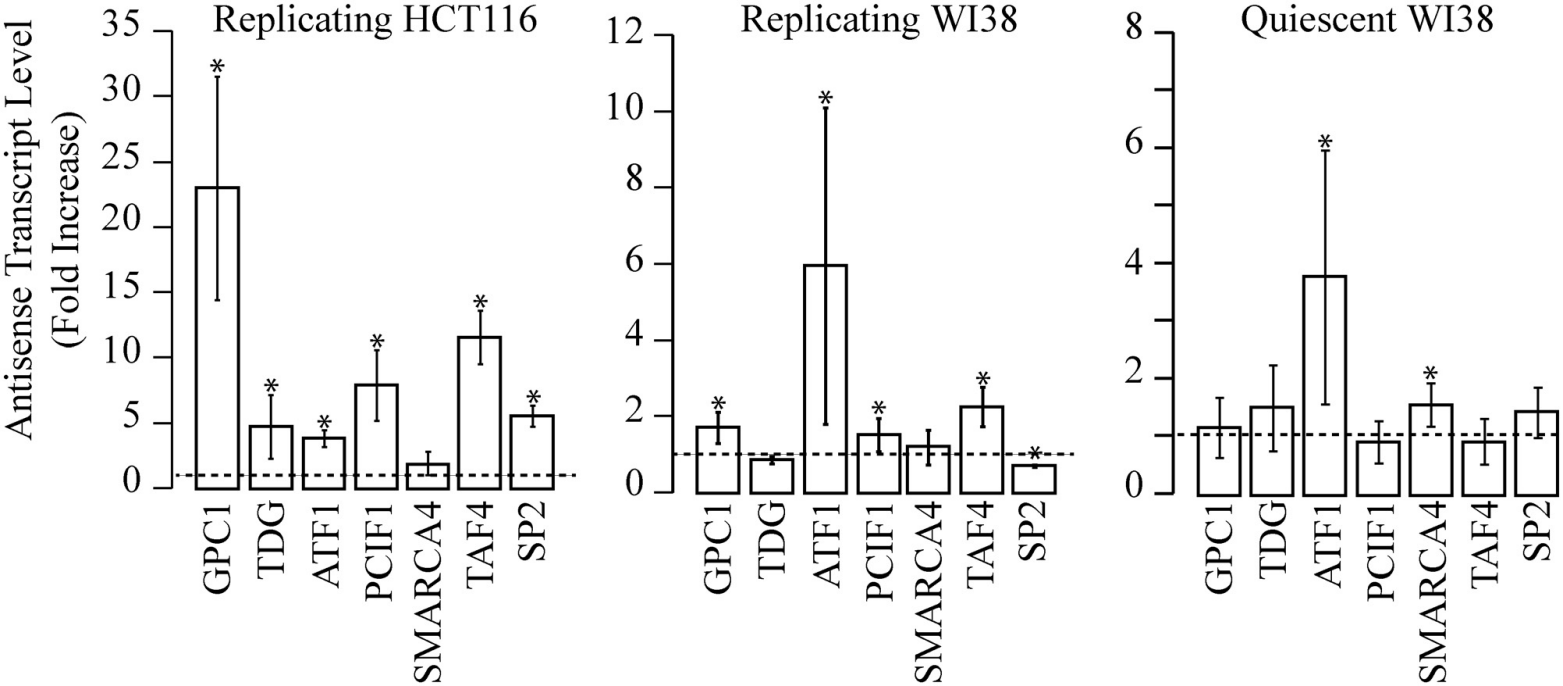
Antisense transcript levels induced by CPT in replicating HCT116 and WI38, and in quiescent WI38 cells. Promoter-associated antisense transcripts were determined by rtqPCR after 4 hours of CPT treatment at 10 μM. PCR determinations were normalized to cytochrome b mRNA and to untreated cells (dotted line). Values are means +/− SEM of two determinations from at least two independent experiments (* *P* <0.05).

To further characterize aRNAs, we asked if their induction was dependent on transcription or replication. Thus, we have first investigated CPT effects in the presence of flavopiridol (FLV), a specific inhibitor of Cdk9 and RNA PolIItranscription. Moreover, we extended the investigation to human fibroblast-like embryonic cells (WI38) at genomic loci selected based on previous findings [6]. Treatments with 10 μM CPT in replicating WI38 cells stimulate antisense accumulation in four out of seven of the selected loci (Fig 4), showing that CPT stimulates antisense transcription in non-cancer cells as well. However, the CPT effects are lower in WI38 than HCT116 cells and the promoter pattern of antisense accumulation is different between the two cell lines.

To evaluate the role of replication on drug effects, we studied the CPT response in serum-starved quiescent WI38 cells (Fig 4, quiescent WI38). Among the studied loci, ATF1 gene promoter accumulates antisense transcripts at the highest levels in both proliferating and resting cells. As in resting cells the enhancement of antisense transcript levels was still present and not significantly different from cycling cells, we suggest that the CPT effect may be independent from DNA replication. The slight reduction between replicating and quiescent cells could be due to a lower transcription rate in the latter. In serum-starved quiescent WI38 cells, we performed a drug dose-response after 1 and 4 hours of treatment (Fig B in S1 File) showing some increase over time. To determine the role of transcription on CPT effects, we then pretreated for 1 hour quiescent WI38 cells with FLV. Under these conditions, no increase of antisense transcript could be detected at the selected loci (Fig C in S1 File), showing that the drug effect is highly dependent on active transcription in resting human cells.

Next, we wondered whether increase of aRNAs was dependent on the cellular response to double-stranded DNA break (DSB) induced by CPT. As transcription-blocking Top1ccs produce DSBs in quiescent WI38 cells in a manner dependent on ATM and DNA-PK[27,41,42], we investigated if the accumulation of aRNAs could be due to DSB-activated kinases, DNA-PK and ATM, after Top1cc stabilization by CPT. Cells were first exposed to ATM or DNA-PK inhibitor for 1 hour, and then CPT was added to the medium for additional 4 hours, in both HCT116 and WI38 cell lines. In all the tested conditions, inhibition of the studied DDR kinases did not significantly alter CPT effects on antisense accumulation (Fig D in S1 File). Therefore, the data suggest that ATM or DNA-PK activation by DSB [41]is not apparently required for CPT effects on aRNAs.

**Fig 5:**
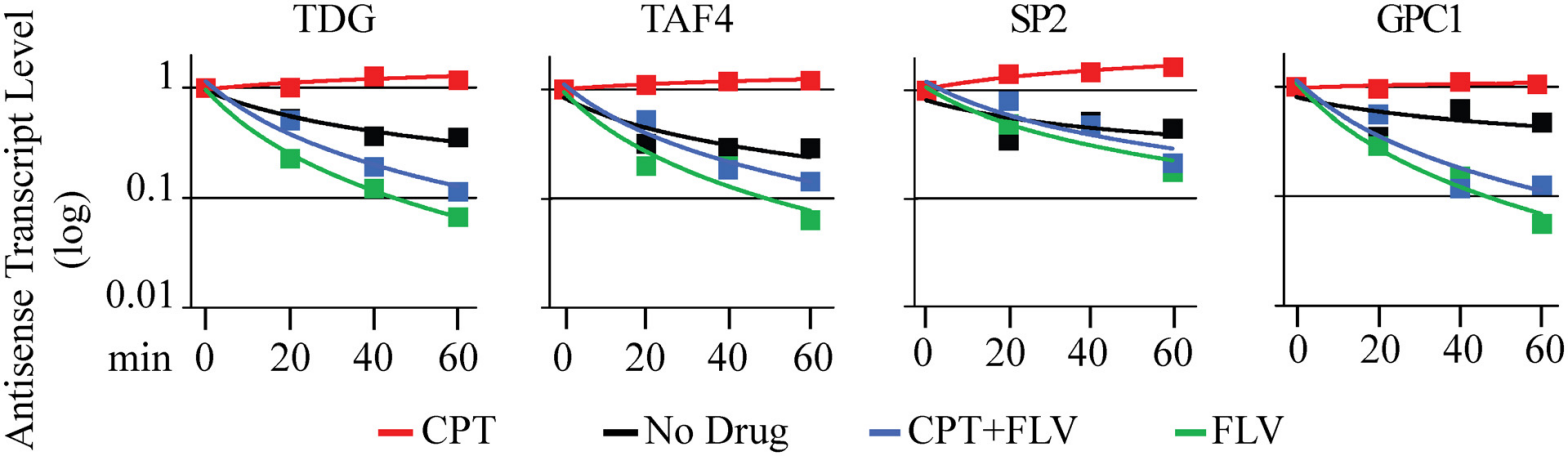
Turnover of aRNAs is influenced by CPT. Cells where firstly stimulated for antisense accumulation by a 4 hours treatment with CPT (time 0). Successively CPT was removed (black lines) or maintained (red lines) in the medium. In addition, FLV was added to block transcription in absence (green lines) and in presence (blue lines) of CPT, and antisense transcript levels determined by rtqPCR after additional 20, 40 and 60 minutes. PCR determinations were normalized to β-actine mRNA and to aRNAs levels at time 0. A representative experiment is here reported.

However, as the above findings do not exclude that CPT-induced aRNA accumulation is part of the cellular response to Top1ccs, we wondered whether CPT could actively modify the turnover of aRNA. Therefore, we treated HCT116 cell with CPT for 4 hours, and then determined the levels of the studied transcripts at different times to evaluate their degradation rates. The experiments were performed adding FLV at the end of a 4 hour period of CPT treatment to block transcription. Under these conditions, the degradation rates of TDG, TAF4, SP2 and GPC1 antisense transcripts (Fig 5 and Fig E in S1 File) are lower in the presence of CPT (compare green vs blue lines) and the transcripts are lost faster in drug-free medium (compare black vs red lines) (Fig 5 and Fig E in S1 File). Thus, the data clearly show that CPT seems to stabilize antisense RNAs, likely preventing their fast removal and degradation.

### CPT determines the accumulation of truncated sense transcripts at 5 repre repre repo CPT (time 0). Successivel

The above findings show that persistent Top1 inhibition by CPT promotes the stabilization of antisense transcripts upstream to the TSS and a general reduction ofantisense R-loops at those loci after long treatment time. As R-loops have been shown to form also downstream the TSS [25,38] (see also Fig 1A), we have then investigated whether CPT induced the accumulation of sense transcripts at the 5’-end of genes as well. We have then mapped paired sequence tags obtained from total cellular RNA depleted of ribosomal RNAs and treated with bisulfite to maintain the information of strand direction [6]. Non overlapping genes were grouped depending on their FPKM in four categories from low to high expression levels, and we focused on gene regions from -2000 bases upstream the TSS to +2000 bases downstream the TES (transcription end site) in both CPT-treated and control HCT116 cells. Then we plotted the distribution of the sense reads along these genes.

**Fig 6:**
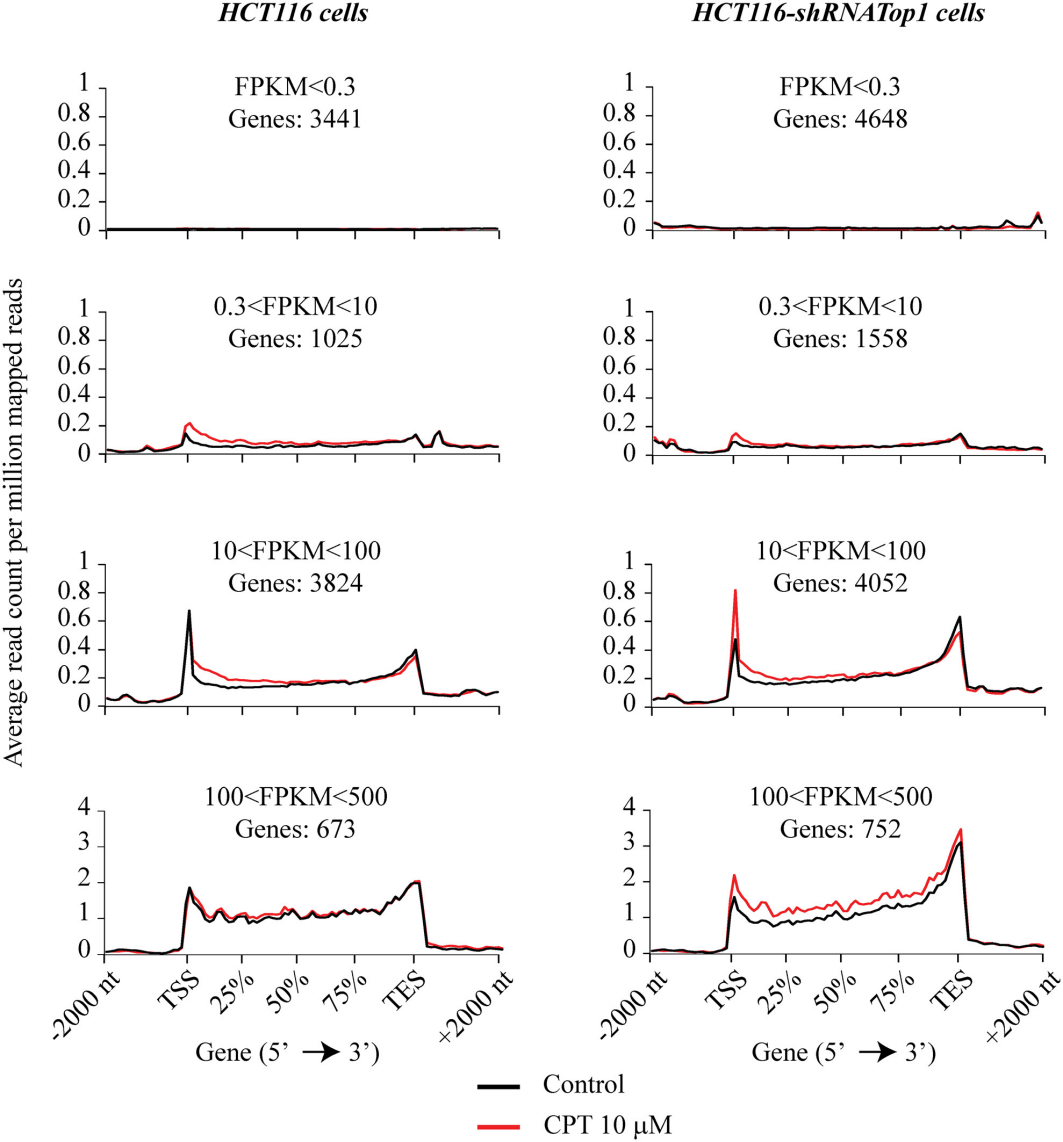
CPT interferes with sense transcription at 5’-end of genes. Sense tags distribution along non-overlapping Refseq genes of HCT116 and HCT116-shRNATop1 cells, were analyzed in a region from 2000 bases upstream the TSS to 2000 bases downstream the TES using NGSplot software. Here are reported genes whose FPKM value is below 0,3 up to 500 divided in four groups. Control reads are reported in black lines and CPT reads in red lines.

Sense tag levels of all non-overlapping genes were clearly dependent on gene expression levels, however they were similar among expressed gene sets (Fig 6). Tag distribution of control cells almost overlapped with that of CPT-treated cells with the exception of the region immediately downstream to the TSS in HCT116 cells (Fig 6). In particular, sense tags were increased at the 5’-end of genes of the two intermediate expression categories following CPT treatment (Fig 6).

**Fig 7:**
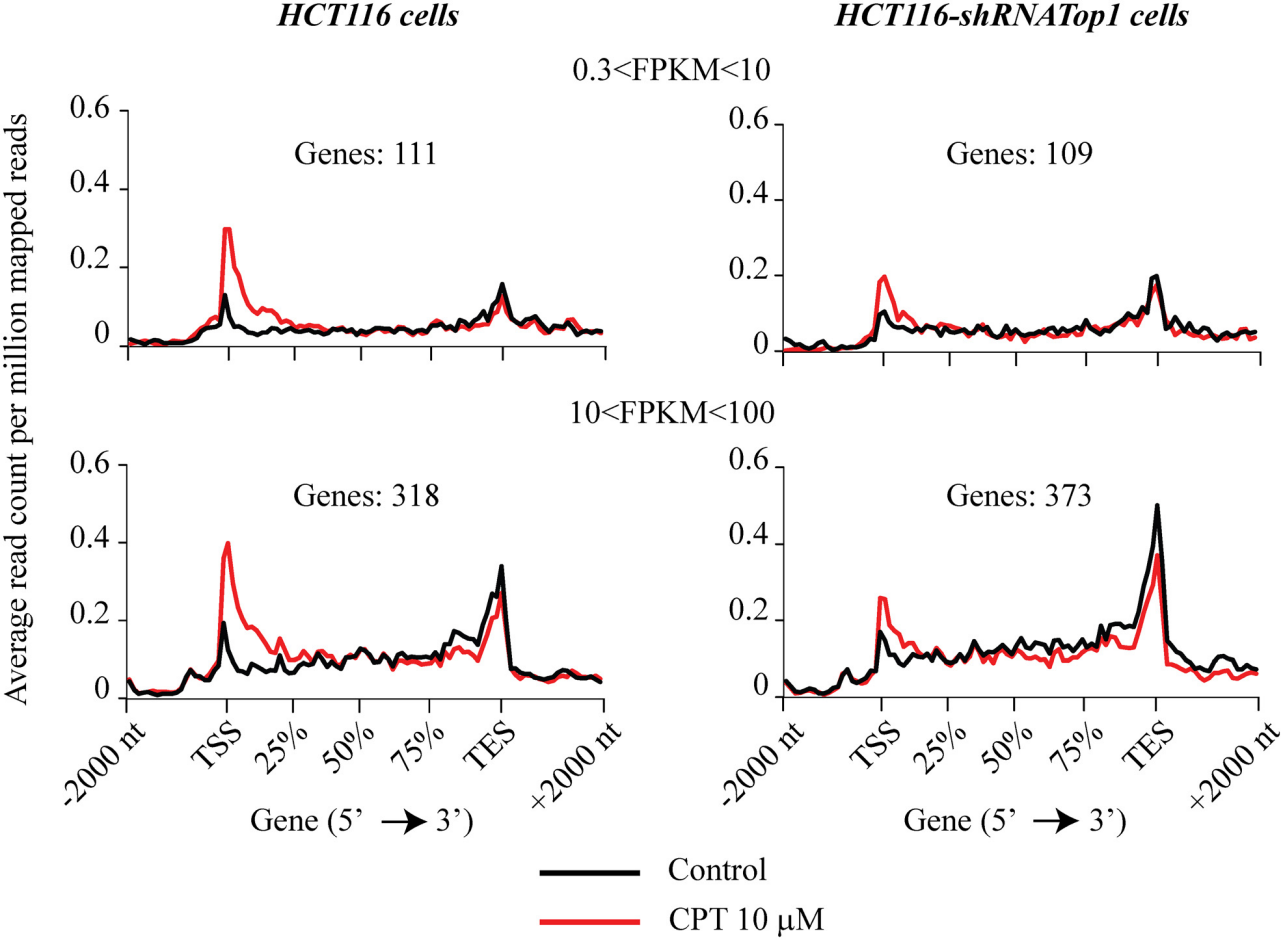
CPT modulates sense transcription in a particular subset of genes. Sense tags distribution along non-overlapping Refseq genes of HCT116 cells and HCT116-shRNATop1 cells, were analyzed in a region from 2000 bases upstream the TSS to 2000 bases downstream the TES using NGSplot software. Here, genes have been divided in two groups based on their FPKM. These genes have been selected for an accumulation of sense reads in the 5’ region (CPT-reads minus Control-reads: 10 ≥ 100) and a reduction of sense reads at the 3’ region (CPT-reads minus Control-reads: ≤ 5). Furthermore, genes selected have a fold change above 2 and a minimum number of sense reads above 5 in the 5’ region of CPT treated sample. Reads were normalized to the length of each region (1000 bp). Control reads are reported in gray dotted line and CPT reads in black line. Black arrows indicate an accumulation or not of reads at 5’ level after CPT treatment (10 μM for 4 hours).

Thus, as the intermediately active genes show such a drug effect, we selected genes showing an increase of sense reads within the first 1000 bases downstream the TSS as well as reduced or equal levels of sense reads 1000 bases upstream the TES (Fig 7). For these genes we observed a marked and specific increase of sense tagsin the two gene sets that was clearly dependent on Top1 (compare HCT116 and HCT116-shRNATop1 cells) (Fig 7), indicating that it was dependent on the cellular level of Top1 in response to CPT (Fig 7). The specific accumulation of sense tags at 5’-end regions was then confirmed at specific loci by rtqPCR(Figs F and G in S1 File). Gene ontology analyses of the two gene sets did not reveal any significant gene attribute enriched in the studied groups suggesting a lack of common functional or structural characteristic.

## Discussion

A fine regulation of Top1 activity at active genes is essential to maintain a proper transcription process as Top1-deficient yeast cells show impaired transcriptional processes and accumulate abortive transcripts by RNA PolI[16]. We have previously demonstrated that Top1 inhibition by CPT leads to unbalanced sense/antisense transcript levels at bidirectional CpG-island promoters [6]. The newly-identified antisense transcripts, with a median size of about 800 bases, are accumulated during drug treatment in a Top1-dependent manner mainly at promoters of intermediate activity. In the present work, we aimed to better characterize these aRNA and to establish a relationship between them and R-loop formation at divergent promoters. Our data show a dynamical response to Top1 inhibition by CPT: aRNAs can form antisense R-loops at promoters in unperturbed N-TERA-2 cells, and immediately following Top1 inhibition by CPT, promoter-associated R-loops can be further stabilized and extended in length at active TSS. This effect is likely due to the formation of Top1ccs close to R-loops structures at promoters, as we showed that Top1ccs are transiently stabilized by CPT at promoter regions [6]. In contrast, persistent CPT inhibition of Top1 markedly reduces R-loop structures and accumulates truncated sense transcripts at 5’ ends of intermediately active genes. Thus, the findings indicate that Top1 may regulate transcription initiation by regulating RNA Pol II-generated negative supercoils, which in turn can favor R-loop formation at promoters, and that transcript accumulation at TSS is a transcriptional response to persistent Top1 poisoning and transcription inhibition. The proposed role of Top1 at promoters is in agreement with previous findings on the effects of Top1 deletion [14,16].

As R-loops can either trigger genome instability or mediate transcription regulation[20,25,43], we have defined whether or not transcriptional stress induced by Top1 inhibition could be mediated by formation of R-loops at active regions. The results show that R-loop can likely form in the antisense orientation at the studied divergent CGI promoters in untreated cells. Interestingly, CPT perturbs R-loops immediately upon addition to the growth medium as short CPT treatments (2–10 minutes) extend or generate new R-loops at the studied promoters whereas the Top1 poison completely abolishes R-loops at most of the studied promoters following longer treatment times. Therefore, it is likely that Top1 inhibition has a direct and rapid effect on R-loop formation favored by excess negative supercoils behind elongating RNA Pol II, which has not be relieved by Top1. This increase is indeed transient as other homeostatic control of DNA superhelicity (for instance, by other DNA topoisomerases) can likely restore default levels of template supercoils. This is in agreement with a recent paper[14] showing that Top1 is most efficiently recruited at promoters of intermediate activity and that a short CPT treatment determines increased negative supercoils upstream and immediately adjacent to the TSS. Such an increase of negative supercoiling of the DNA template would likely favor both stabilization and extention of R-loop structures at active promoters.

Our findings also show that when Top1 is persistently inhibited by CPT then R-loop formation is also reduced at promoters of active genes. As R-loop levels are dependent on active transcription, then persistent CPT treatments, which are known to strongly inhibit transcription elongation[44], may preclude the formation of R-loops. Nevertheless, CPT may have more dynamic effects on R-loops in relation to their genomic location, as we observed that R-loops persist even after 4 hour of CPT treatment at TAF4 promoter. In postmitotic neurons, persistent CPT treatments have been shown to affect cell viability [45]and induce transcriptional DSBs in an R-loop-dependent manner[27]. Thus, it can be speculated that R-loops formed at specific genomic location may be stabilized by CPT leading to irreversible double-strand breakage and apoptosis. Here, we have attempted to evaluate if the increase of antisense RNAs at promoters is downstream to DDR pathway activated by CPT, and our findings show that antisense transcript increase is not simply related to either ATM or DNA-PK activation in HCT116 and WI38 human cells. However, it remains to be established whether DSBs themselves or other DDR proteins or a combination of both may be implicated in aRNA induction in response to CPT.

The increase of antisense and sense transcripts may in principle be originated by an enhanced synthesis and/or a reduced degradation of them. We previously reported that CPT still determines a similar increase rate of promoter-associated transcripts when transcription was inhibited by DRB as compared with the absence of DRB [6], suggesting that an enhanced synthesis of antisense RNA is unlikely even if we cannot rule out completely this possibility. In agreement with these data, the present findings show that CPT impairs degradation of antisense transcripts. As exosome silencing has been shown to increase the levels of cryptic antisense RNA at promoters [46], our findings suggest that exosome activity may be somewhat reduced in cells treated with CPT.

The present findings have established that aRNAs can form antisense R-loops at the studied divergent promoters, in agreement with recent published data [47]. In addition, our findings show that Top1 inhibition by CPT have dynamic and site-specific effects on R-loop structures. In particular, immediately upon addition, CPT can favor R-loop formation whereas, at longer time of treatment, CPT markedly reduces R-loop levels. Interestingly, R-loops persist at certain active promoters and other genomic loci along with a more general induction of truncated sense and antisense RNAs at active TSS. The findings define new aspects of the specific CPT effects at transcriptional levels in human cancer and normal cells.

## Supporting Information

S1 File **Fig A**, *Representative experiment of DRIVE Assay in* RNaseH1 pretreated (black bars) and not pretreated (white bars) samples. (Panel A) Three positive (RPL13A, BTBD19, MYADM) and two negative (SNRPN, a-SAT) loci for R-loop formation as previously reported in Ginno et al. 2012 [25]. (Panel B) Eight divergent promoters selected on the basis of their ability to show increase of antisense transcripts after Top1 inhibition by CPT. TNIK here is a negative control. **Fig B**, Promoter-associated antisense transcripts were evaluated by rtqPCR after 1 and 4 hours of CPT treatment at different doses (2, 5 and 10 μM). PCR determinations were normalized to cytochrome b mRNA and to untreated cells (dotted line). Values are means +/− SEM of at two determinations of at least two independent experiments. **Fig C**, Promoter-associated antisense transcripts were evaluated by rtqPCR after 4 hours of CPT treatment (10 μM) in presence of FLV (gray bars). PCR determinations were normalized to cytochrome b mRNA and to untreated cells (dotted line). Values are means +/− SEM of at two determinations of at least two independent experiments. **Fig D**, Promoter-associated antisense transcripts were evaluated by rtqPCR after 4 hours of CPT treatment (10 μM) in presence of ATM (gray bars) and DNA-PK (white bars) inhibitors. PCR determinations were normalized to cytochrome b mRNA and to untreated cells (dotted line). Values are means +/− SEM of at two determinations of at least two independent experiments. **Fig E**, Cells where firstly stimulated for antisense accumulation by a 4 hours treatment with CPT (time 0). Successively CPT was removed (black lines) or maintained (red lines) in the medium. In addition, FLV was added to block transcription in absence (green lines) and in presence (blue lines) of CPT, and antisense transcript levels determined by rtqPCR after additional 20, 40 and 60 minutes. PCR determinations were normalized to β-actine mRNA and to aRNAs levels at time 0. Here is reported a representative experiment different from the one reported in Fig 5. **Fig F**, The accumulation of sense transcripts in the 5’ region and the reduction of sense transcripts in 3’ region of selected genes were determined by rtPCR in the HCT116 cells and were evaluated after treatment of the indicated cell lines with CPT 10 μM for 4h. The selected genes showed a CPT-increased in the 5’ region and a reduction in the 3’ region tag clusters as determined with RNA-seq data, with the exception of KLHL22 gene that had a sense transcript reduced by CPT. PCR determinations were normalized to cytochrome b mRNA and to untreated cells (dotted line). Values are means ± SEM of at least four determinations of six independent experiments for each panel. **Fig G**, The accumulation of sense transcripts in the 5’ region and the reduction of sense transcripts in 3’ region of selected genes were determined by rtPCR in the HCT116-shRNATop1 cells and were evaluated after treatment of the indicated cell line with CPT 10 μM for 4h. The selected genes showed a lower CPT-increased in the 5’ region and a lower reduction in the 3’ region tag clusters as determined with RNA-seq data, with the exception of AEN gene that had a higher sense transcript increased by CPT, taking into account the HCT116 cells line. PCR determinations were normalized to cytochrome b mRNA and to untreated cells (dotted line). Values are means ± SEM of at least four determinations of two independent experiments.
(PDF)
