# Supplementary material for: Dynamic Effects of Topoisomerase I Inhibition on R-Loops and Short Transcripts at Active Promoters
Source: PLoS One. 2016 Jan 19;11(1):e0147053. doi: 10.1371/journal.pone.0147053 (PMC4718701; doi:10.1371/journal.pone.0147053)
Supplement: S1 File — Fig A, Representative experiment of DRIVE Assay in RNaseH1 pretreated (black bars) and not pretreated (white bars) samples. (Panel A) Three positive (RPL13A, BTBD19, MYADM) and two negative (SNRPN, a-SAT) loci for R-loop formation as previously reported in Ginno et al. 2012 [25]. (Panel B) Eight divergent promoters selected on the basis of their ability to show increase of antisense transcripts after Top1 inhibition by CPT. TNIK here is a negative control. Fig B, Promoter-associated antisense transcripts were evaluated by rtqPCR after 1 and 4 hours of CPT treatment at different doses (2, 5 and 10 μM). PCR determinations were normalized to cytochrome b mRNA and to untreated cells (dotted line). Values are means +/− SEM of at two determinations of at least two independent experiments. Fig C, Promoter-associated antisense transcripts were evaluated by rtqPCR after 4 hours of CPT treatment (10 μM) in presence of FLV (gray bars). PCR determinations were normalized to cytochrome b mRNA and to untreated cells (dotted line). Values are means +/− SEM of at two determinations of at least two independent experiments. Fig D, Promoter-associated antisense transcripts were evaluated by rtqPCR after 4 hours of CPT treatment (10 μM) in presence of ATM (gray bars) and DNA-PK (white bars) inhibitors. PCR determinations were normalized to cytochrome b mRNA and to untreated cells (dotted line). Values are means +/− SEM of at two determinations of at least two independent experiments. Fig E, Cells where firstly stimulated for antisense accumulation by a 4 hours treatment with CPT (time 0). Successively CPT was removed (black lines) or maintained (red lines) in the medium. In addition, FLV was added to block transcription in absence (green lines) and in presence (blue lines) of CPT, and antisense transcript levels determined by rtqPCR after additional 20, 40 and 60 minutes. PCR determinations were normalized to β-actine mRNA and to aRNAs levels at time 0. Here is reported a representativ [file pone.0147053.s001.pdf]

SUPPLEMENTARY INFORMATIONS

Dynamic Effects of Topoisomerase I Inhibition on R-loops and Short Transcripts at Active Promoters.

Marinello Jessica, Bertoncini Stefania, Aloisi Iris, Cristini Agnese, Malagoli Tagliazucchi Guidantonio, Forcato Mattia, Sordet Olivier and Capranico Giovanni

SUPPLEMENTARY FIGURES OF S1 FILE.

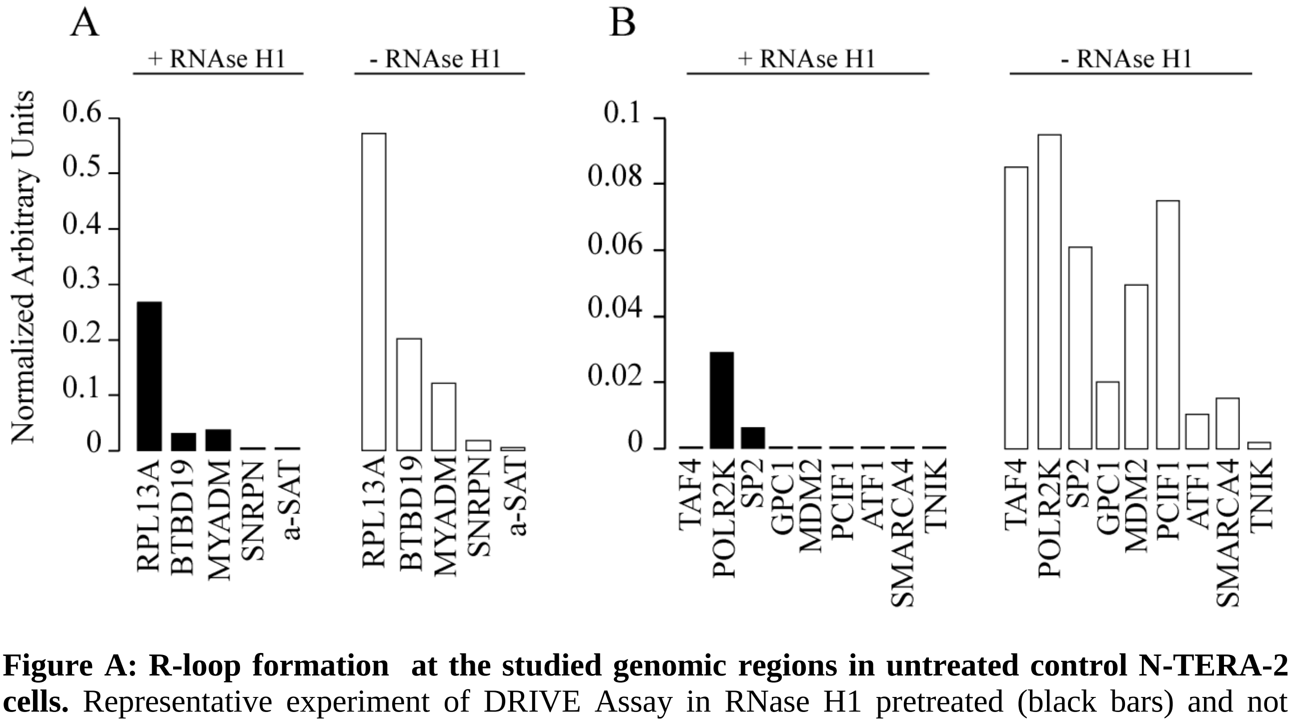

Figure A: R-loop formation at the studied genomic regions in untreated control N-TERA-2 cells. Representative experiment of DRIVE Assay in RNase H1 pretreated (black bars) and not pretreated (white bars) samples. (A) Three positive (RPL13A, BTBD19, MYADM) and two negative (SNRPN, a-SAT) loci for R-loop formation as previously reported in Ginno et al. 2012 [25]. (B) Eight divergent promoters selected on the basis of their ability to show increase of antisense transcripts after Top1 inhibition by CPT. TNIK here is a negative control.

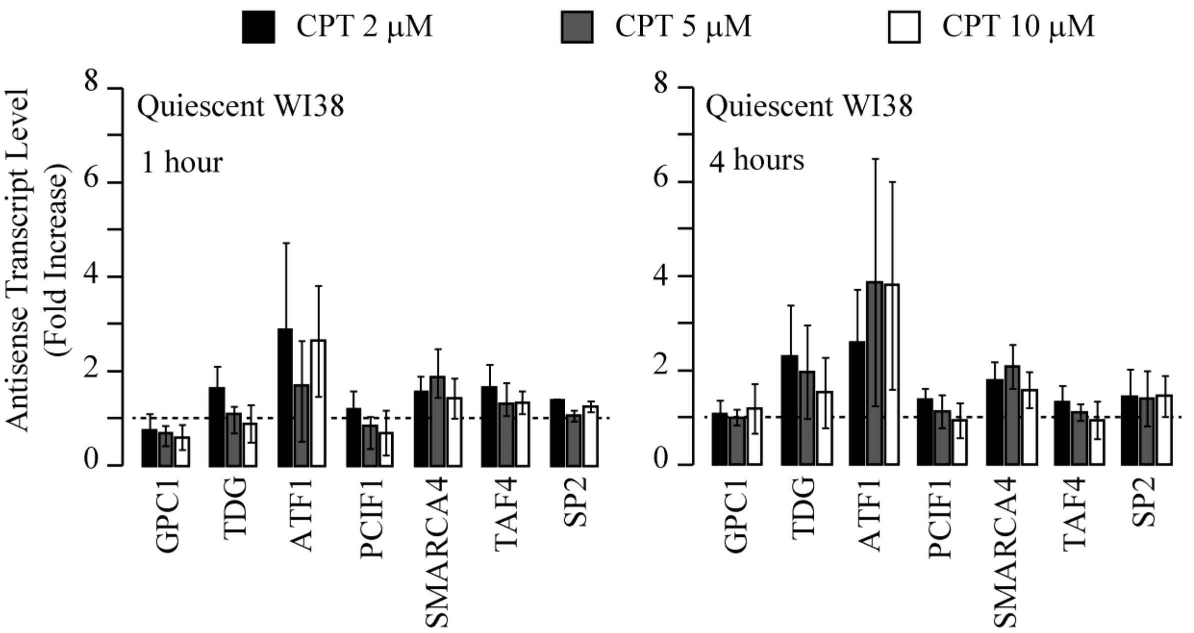

Figure B: Time course and dose response in quiescent WI38 cells. Promoter-associated antisense transcripts were evaluated by rtqPCR after 1 and 4 hours of CPT treatment at different doses (2, 5 and 10 μM). PCR determinations were normalized to cytochrome b mRNA and to untreated cells (dotted line). Values are means +/− SEM of at two determinations of at least two independent experiments.

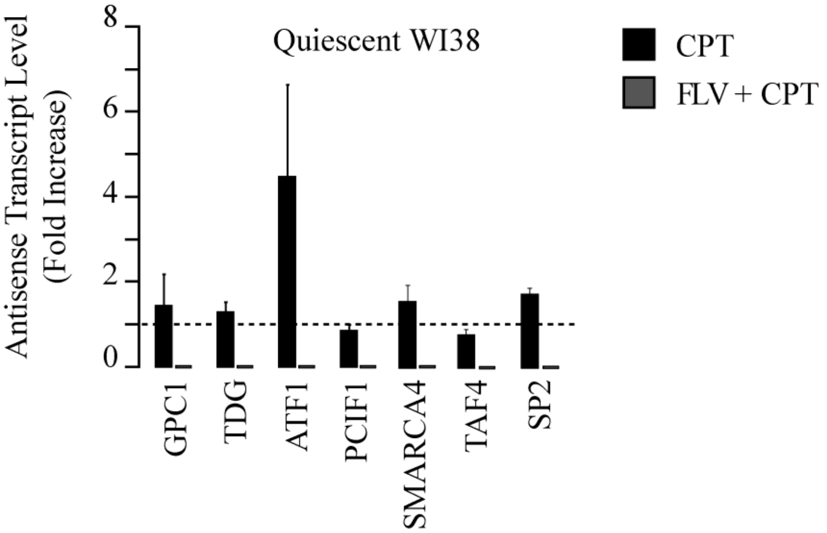

Figure C: Effect of FLV treatment on antisense transcription. Promoter-associated antisense transcripts were evaluated by rtqPCR after 4 hours of CPT treatment (10 μM) in presence of FLV (gray bars). PCR determinations were normalized to cytochrome b mRNA and to untreated cells (dotted line). Values are means +/− SEM of at two determinations of at least two independent experiments.

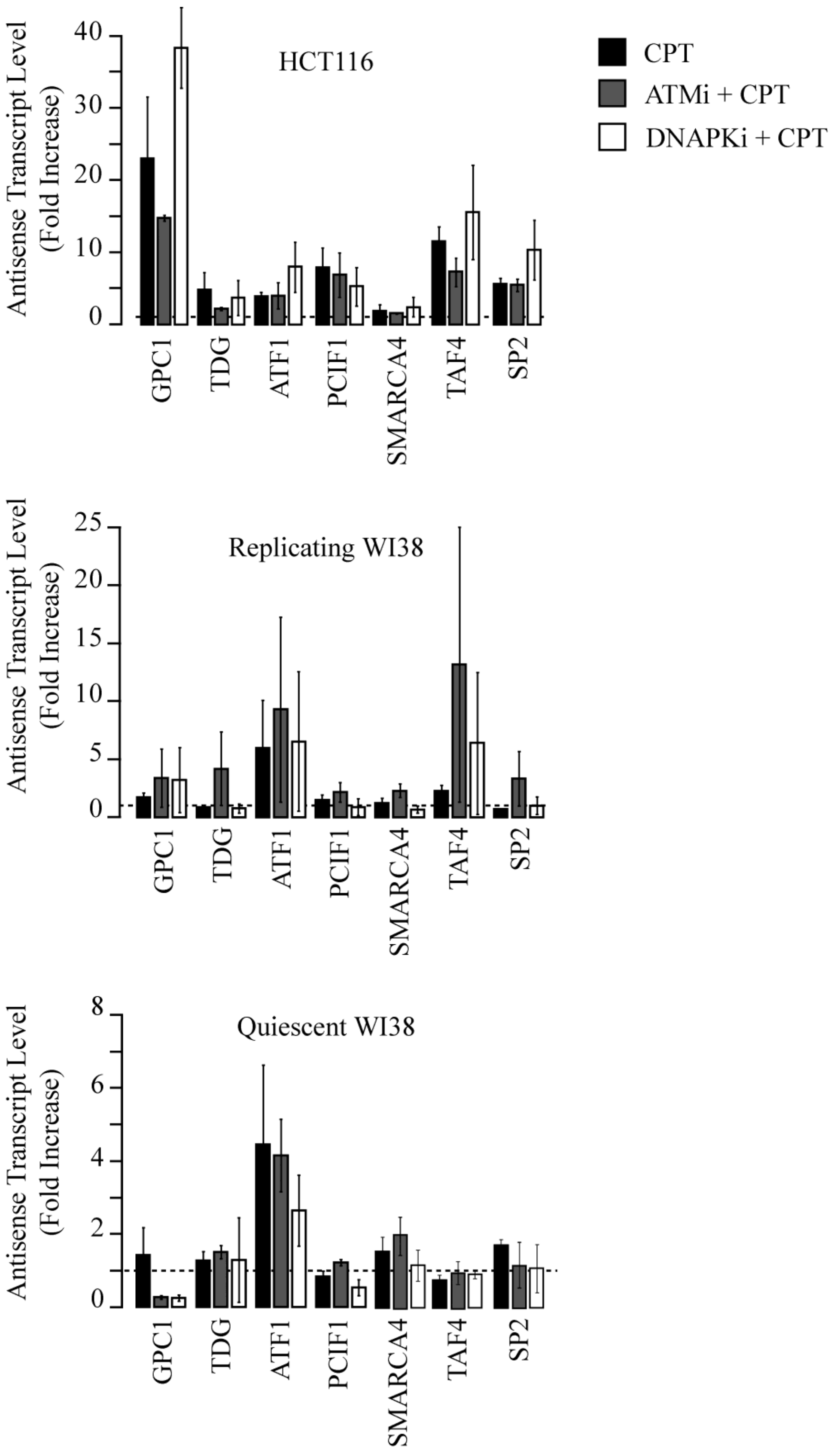

Figure D: DDR activation effect on antisense transcription. Promoter-associated antisense transcripts were evaluated by rtqPCR after 4 hours of CPT treatment (10 μM) in presence of ATM (gray bars) and DNA-PK (white bars) inhibitors. PCR determinations were normalized to cytochrome b mRNA and to untreated cells (dotted line). Values are means +/− SEM of at two determinations of at least two independent experiments.

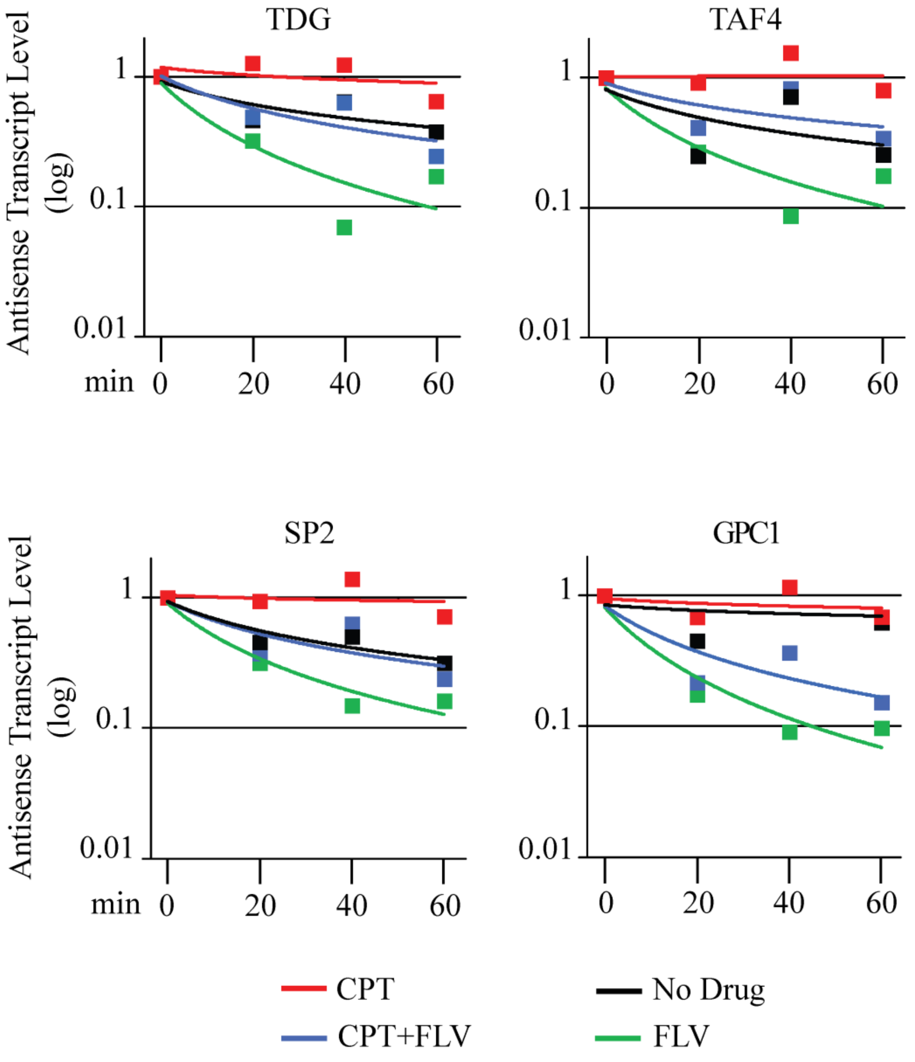

Figure E: Degradation rates of aRNAs. Cells where firstly stimulated for antisense accumulation by a 4 hours treatment with CPT (time 0). Successively CPT was removed (black lines) or maintained (red lines) in the medium. In addition, FLV was added to block transcription in absence (green lines) and in presence (blue lines) of CPT, and antisense transcript levels determined by rtqPCR after additional 20, 40 and 60 minutes. PCR determinations were normalized to β-actine mRNA and to aRNAs levels at time 0. Here is reported a representative experiment different from the one reported in figure 5.

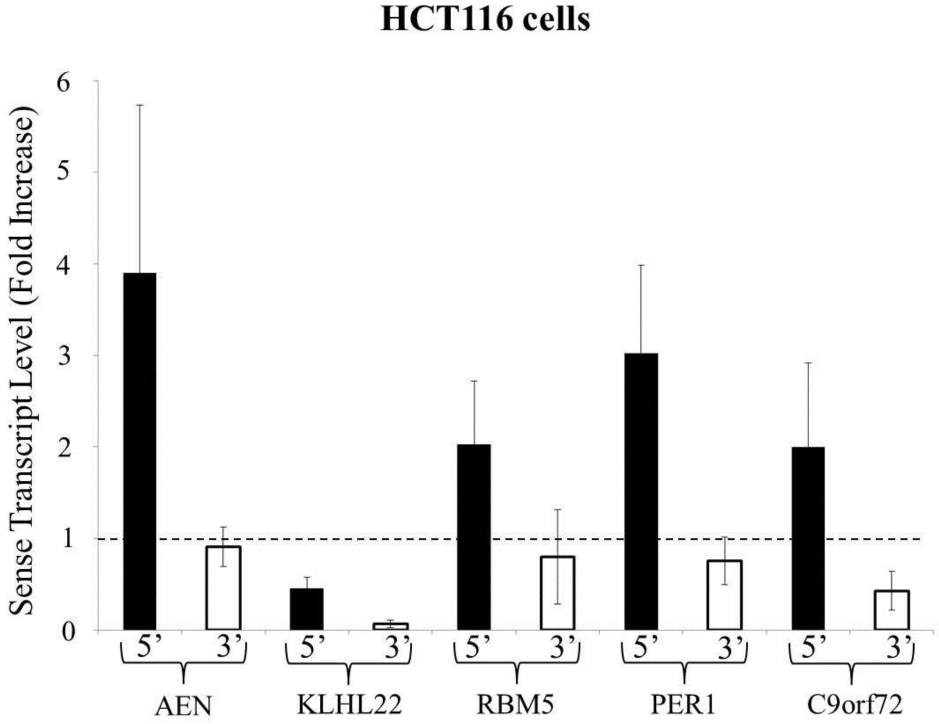

Figure F: CPT interference with sense transcription. The accumulation of sense transcripts in the 5’ region and the reduction of sense transcripts in 3’ region of selected genes were determined by rtPCR in the HCT116 cells and were evaluated after treatment of the indicated cell lines with CPT 10 µM for 4h. The selected genes showed a CPT-increased in the 5’ region and a reduction in the 3’ region tag clusters as determined with RNA-seq data, with the exception of KLHL22 gene that had a sense transcript reduced by CPT. PCR determinations were normalized to cytochrome b mRNA and to untreated cells (dotted line). Values are means ± SEM of at least four determinations of six independent experiments for each panel.

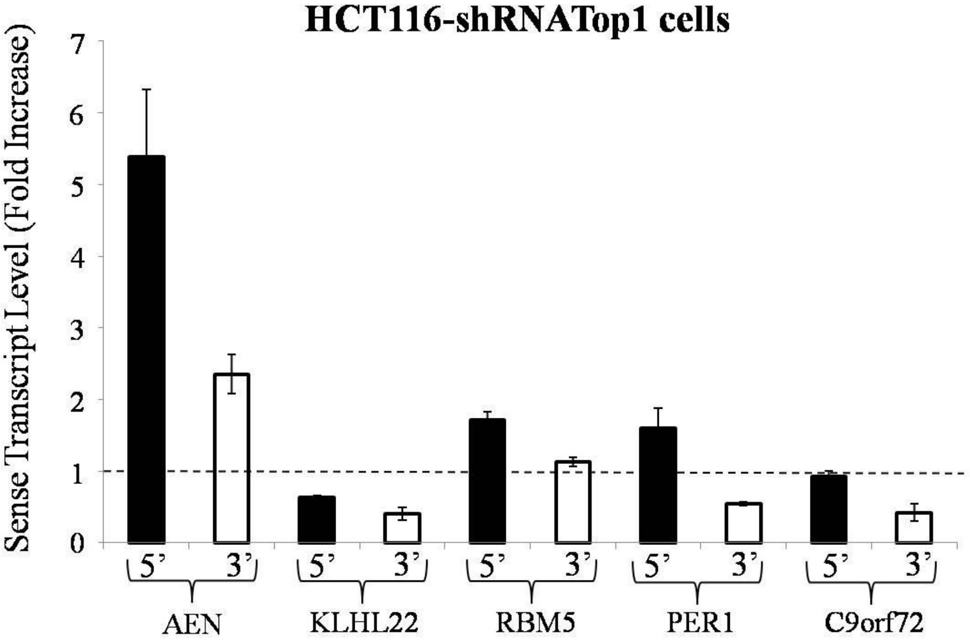

Figure G: CPT interference with sense transcription. The accumulation of sense transcripts in the 5' region and the reduction of sense transcripts in 3' region of selected genes were determined by rtPCR in the HCT116-shRNATop1 cells and were evaluated after treatment of the indicated cell line with CPT 10 µM for 4h. The selected genes showed a lower CPT-increased in the 5' region and a lower reduction in the 3' region tag clusters as determined with RNA-seq data, with the exception of AEN gene that had a higher sense transcript increased by CPT, taking into account the HCT116 cells line. PCR determinations were normalized to cytochrome b mRNA and to untreated cells (dotted line).Values are means ± SEM of at least four determinations of two independent experiments.
